# Common Cognitive Biases in Nephrology Critical Care: A Plea for Metacognition

**DOI:** 10.7759/cureus.6304

**Published:** 2019-12-06

**Authors:** Gurkeerat Singh, Hirsh Sharma, Jean-Sebastien Rachoin, Sharad Patel

**Affiliations:** 1 Critical Care, Cooper University Hospital, Cooper Medical School, Camden, USA; 2 Critical Care, Cooper University Hospital, Camden, USA

**Keywords:** critical care

## Abstract

The intensive care unit (ICU) is an incredibly complex environment, and ICU rounds are mentally taxing. The cognitive biases that tend to arise in mentally taxing environments such as the ICU pose a risk to patients. This review discusses 10 common cognitive biases and logical fallacies using examples in Nephrology Critical Care. Our objective is to promote metacognition (i.e., an awareness of one’s cognition) among physicians. A state of metacognition is not a panacea, but aspiring for metacognition allows the critical care physician to improve chances for optimal patient outcomes.

## Introduction and background

“What then in the last resort are the truths of mankind?--They are the irrefutable errors of mankind.” - Friedrich Nietzsche [1].

Critical thinking is a metabolically expensive process. To conserve precious energy and to promote expediency, the mind creates mental short cuts called heuristics [2]. Heuristics serve us well in most aspects of life, but they can lead to systematic errors in reasoning called cognitive biases, which, when applied inappropriately, can lead to enormous consequences. The higher the cognitive load, the more predisposed the mind is to cognitive biases and errors in logic [2]. The intensive care unit (ICU) is an incredibly complex environment, and to say that intensivists are cognitively loaded would be an understatement. ICU rounds are mentally taxing. Therefore, we must recognize that we have a predisposition to cognitive biases, which could be detrimental to our patients. This review describes 10 common cognitive biases and logical fallacies through the prism of Nephrology Critical Care with the goal of promoting metacognition (i.e., an awareness of one’s cognition). Aspiring for metacognition is not a panacea, but the first step of alleviating a problem is to recognize the existence of it.

## Review

Table 1 presents a tabular overview of the 10 cognitive biases and logical fallacies discussed in this review.

**Table 1:** Ten cognitive biases and logical fallacies ACS, abdominal compartment syndrome; AKI, acute kidney injury; CHF, congestive heart failure; COPD, chronic obstructive pulmonary disease; echo, echocardiography; HRS, hepatorenal syndrome; HTN, hypertension; IAH, intraabdominal hypertension; IAP, intraabdominal pressure; IVC, inferior vena cava; RBF, renal blood flow; RV, renal vein.

Musing	Fallacy/Bias	Describing the fallacy/bias	Example
Rise in creatinine	Post hoc fallacy	After this, therefore, because of this	Dr. S stops furosemide after a bump in creatinine levels. Furosemide was initiated 6 hours previously.
Hepatorenal syndrome	Base rate fallacy	Base rate neglect	Patient A with a history of cirrhosis presents with fever, cough, and evidence of AKI. Dr. G suspects HRS.
Abdominal Compartment Syndrome	False dichotomization	falsely claimed to be either/or situation	Dr. K is relieved to hear that the IAP is 18, no management changes made since it is not ACS.
Normal saline	Status quo bias	Opting for the familiar	Dr. M orders normal saline for all patients despite the accessibility of more balanced crystalloids. “Its what I’ve always done.”
Lactic acidosis	Reduction fallacy	Oversimplification of a single cause	Patient G with a history of COPD presents with pneumonia, receiving albuterol which leads to a lactate rise. Dr. A reflexively administers multiple fluid boluses.
Furosemide-related hypernatremia	Law of instrument bias or Law of the hammer	Over-reliance on a familiar tool	Patient G presents with CHF exacerbation, requires intubation. The patient becomes agitated while getting furosemide; serum sodium is noted to be 155 mmol/L.
Shock precluding fluid removal	Semmelweis reflex bias	The tendency to reject new evidence in the face of a known paradigm	Patient G has history pulmonary HTN, echo with evidence of a dilated RV/IVC. IAH present. On a small amount of levophed, team not comfortable with diuresis.
Sepsis-related AKI	Illusory truth effect	The tendency to believe information which is repeated to be true	Patient B presents with septic shock and has received 30 cc/kg of crystalloid. Creatinine level rises, Dr. S gives more fluids despite hearing that RBF is increased in sepsis.
Volume tolerance	Automation bias	Algorithmic care trumps clinical judgment	Patient S presents with hypotension with evidence of ALI, bedside echo concerning for RV volume overload. Doctor S gives 30 cc/kg due to suspicion for sepsis.

Creatinine rise: post hoc fallacy (after this, therefore, this)

The diagnosis of acute kidney injury (AKI) based on KDIGO (Kidney disease: Improving Global Outcomes) criteria is dependent on an increase in serum creatinine and reductions in urine output [3]. However, in practice, once there is an insult that results in an acute drop in glomerular filtration rate (GFR), there will be a significant lag in creatinine rise. Creatinine kinetic models have demonstrated that plasma creatinine increases several days after ischemic insults. The creatinine rise will not happen until a steady-state between production and clearance is achieved [4]. The fallacy most often committed in response to perturbations of creatinine is the post hoc fallacy (i.e., after this, therefore, this). Let us consider a common clinical example of this fallacy. A patient’s creatinine increases six hours following the initiation of furosemide. The furosemide treatment gets discontinued as temporal proximity is thought to equate with causality. The clinician must understand that once the creatinine change is appreciated, determination of the renal insult should be systematic, but interventions started a few hours prior are unlikely to be the initial cause of the GFR reduction. Therefore, a temporal analysis of potential renal insults should be made based on an understanding of creatine kinetics, thereby avoiding the post hoc fallacy.

Hepatorenal syndrome: base rate fallacy (frequency of differential does not match base rate)

AKI is a frequent complication of advanced cirrhosis, and hepatorenal syndrome (HRS) is an important differential to consider, but the diagnosis should be discussed with an understanding of its base rate. The primary mechanism for HRS is portal hypertension-related splanchnic vasodilatation with an associated drop in effective circulating volume. AKI with cirrhosis is often assumed to be HRS due to an overestimation of the base rate of HRS. HRS can be diagnosed only after ruling out alternative causes, including intravascular depletion, obstruction, medications, and glomerular disease. The diagnostic criteria for HRS include no improvement in renal function following two days of albumin infusions with complete withdrawal of all diuretics, the exclusion of nephrotoxic agents, and parenchymal renal disease [5]. In an observational analysis of 562 patients with cirrhosis, HRS was found to be the cause in only 13% of the patients, while the infection or intravascular depletion comprise the etiology in 78% of patients [6]. HRS remains a diagnosis of exclusion, and the discrepancy between the actual and perceived base rates should be noted.

Abdominal compartment syndrome: false dichotomization (the fallacy of black and white)

Intraabdominal hypertension (IAH) is defined by an intraabdominal pressure (IAP) of >12 mmHg, which occurs as a result of reduced abdominal compliance. Abdominal compartment syndrome (ACS) is diagnosed when the IAP is > 20 mmHg with concurrent end-organ dysfunction [7]. Renal dysfunction can occur at IAP as low as 12 mmHg due to renal vein compression, arterial vasoconstriction, activation of the renin-angiotensin-aldosterone system, and compression of the renal parenchyma, all of which lead to a reduced filtration gradient [8]. Oliguria may be the first sign before a creatinine rise (see “Creatinine rise: post hoc fallacy”). The numbers-based guidelines lead to a false dichotomization whether the diagnosis is present or not, leaving very little room for gradation. To reintroduce gradation, let us examine the abdominal perfusion pressure (APP)-the difference between mean arterial pressure (MAP) and IAP. Studies have demonstrated that APP values > 60 mmHg are associated with improved outcomes [8]. In the case of a low MAP, an IAP of < 20 mmHg would not meet the criterion for ACS, but there will likely be end-organ dysfunction. The clinician should avoid the dogmatism of threshold medicine-let us not falsely dichotomize complex disease processes.

Normal saline: status quo bias (the bias of opting for the familiar)

Chloride in extracellular fluid is essential in the maintenance of osmolality, acid-base balance, and sodium regulation, but iatrogenic hyperchloremia can be detrimental. Normal saline is the most widely used fluid composition in medicine but has significantly higher chloride load as compared to serum. The evidence favors balanced chloride solutions. Multiple clinical trials have demonstrated that the use of chloride-rich crystalloid solutions is associated with increased risk of AKI and mortality [9], yet its use remains pervasive, likely partially explained by the status quo bias [10]. The physiologic argument against chloride-rich solutions is based on their association with worsening non-anion gap acidemia and decreased renal perfusion [11]. Furthermore, there is little to no detrimental economic impact in switching from a Ringer's lactate solution from normal saline. In fact, given the pervasive use of fluids and the potential reduction in mortality and AKI, there is a likely economic benefit [11]. The physiologic, empiric, and economic arguments for the abandonment of normal saline in the ICU is strong. Therefore, we must move beyond the status quo. As it turns out, normal saline is not so normal.

Lactic acidosis: reduction fallacy (the fallacy of reducing complexity to a single cause)

Lactate production and clearance is a complicated process with many etiologies, but a rise is often met with a fluid bolus. Reflexive fluid administration in response to a lactate rise is an example of the reduction fallacy-the fallacy of reducing a multifaceted process to a single cause-in this case, hypoperfusion. It stands to reason that if lactate increase is a specific marker for volume depletion, volume should be the answer. But how accurate is it? The belief that hypoperfusion is a primary mechanism for lactate rises in sepsis has come under scrutiny. There are physiologic studies that question the perception that reduced oxygen delivery due to hypoperfusion in sepsis causes lactate rise [12]. Adrenergic drive during sepsis increases glycolytic flux, which will produce more lactate, which, during non-stressed states, will be converted back to pyruvate and shunted into the Krebs cycle. Glycolysis outpaces the more distal processes of energy production, such as the Krebs cycle and oxidative phosphorylation; therefore, despite adequate oxygen, lactate levels will rise [13]. Lactic acidosis is a complicated biochemical process that has become victim to the reduction fallacy. 

Furosemide-related hypernatremia: the law of the instrument bias (using one tool for all situations)

Furosemide is a diuretic that works at the loop of Henle and is typically used to achieve negative fluid balances in settings of organ congestion. Where diuresis is concerned, furosemide falls victim to the law of the instrument fallacy. Monotherapy loop diuretic strategy leads to more electrolyte reabsorption in the distal segments of the nephron, leading to more hypotonic urine, which predisposes the patient to hypernatremia. Hypernatremia is one of the more frequent electrolyte disturbances seen in the ICU [14], commonly seen in the setting of diuresis with the improper replacement of free water. Hypernatremia can complicate the ICU course by increasing cardiac dysfunction, agitation, and delirium [14]. Free water in the form of flushes and dextrose in water is given in response to rising sodium, but using a multi-diuretic strategy can reduce rise of serum sodium levels. Using a loop and thiazide diuretic concurrently (with appropriate dose adjustment) will allow for greater sodium excretion as compared to free water, which can prevent hypernatremia while decreasing organ decongestion [14]. Thus, a multi-diuretic approach to organ decongestion may minimize the risk of hypernatremia, reducing free water excretion by increasing natriuresis. Let us not fall prey to the law of the instrument bias for diuresis. 

Shock precluding fluid removal: Semmelweis reflex bias (tendency to reject new evidence in the face of a known paradigm)

Vasoactive medications (e.g., vasopressors and inotropes) are used to treat poor tissue perfusion as indicated by low MAP, oliguria, and low capillary refill. Once vasoactive medicines are in use, particularly in cases of septic shock, the decreased MAP is thought to occur at least partially from hypovolemia [15]. However, critically ill patients in the ICU are only volume responsive approximately 50% of the time [15]. Volume responsiveness physiology can be used to determine the inverse via the following question: “If I remove fluid, will this patient become increasingly hypotensive?” This is a physiologic concept called preload dependence [15]. Echocardiographic signs of a lack of volume responsiveness have shown a reduced incidence of intradialytic hypotension [15]. Preload dependence can be assessed using a non-invasive cardiac monitor, echocardiography, or, rarely, a pulmonary artery catheter. Volume overload is pervasive in septic shock patients [16], and late goal-directed fluid removal should be considered in these patients. Fluid removal in shock violates dogma that is likely maintained by the Semmelweis reflex bias-the rejection of new evidence if it is at odds with the old paradigm. Furthermore, nuance should be preserved in patient care. For example, patients with baseline comorbidities such as pulmonary hypertension may benefit from early fluid removal [16]. Vasoactive medications should not preclude fluid removal in a volume overloaded patient.

Sepsis-related AKI: illusory truth effect (truth is what is repeated)

There is a high incidence of sepsis-related AKI [17]. Early volume expansion remains an important facet of sepsis management, and traditional thought is that the increased renal flow may treat or prevent sepsis-related AKI [17]. The typical mental construct of sepsis-related AKI is of a flow-starved kidney; physiologic studies have not confirmed this paradigm [17]. Induced sepsis in the animal model suggests an increase in renal blood flow, with intrarenal shunting and microcirculatory dysfunction [17], but despite increased knowledge of this phenomenon, we fall prey to the illusory truth effect (i.e., if something is consistently repeated, it must be true). It is crucial to treat intravascular depletion as this can lead to AKI, but volume overload can be similarly detrimental and may lead to the maintenance of kidney impairment in the critically ill [18]. The kidney is an immensely complicated organ. Therefore, the diagnosis and management of AKI should have a corresponding level of critical analysis. We must maintain a skeptic’s eye on repeated “truths.”

Contrast-induced AKI: conservatism bias (inadequate revision of belief when presented with new evidence)

Contrast-induced nephropathy (CIN) is defined as an increase in creatinine of 0.3 mg/dl within 48 to 72 hours to exposure [19]. Implied within the definition of CI-AKI is causality, but a growing body of evidence suggests the correlation is weak at best [18]. Since the first description of CIN [19], the most common form of contrast used is markedly less nephrotoxic than earlier iterations, but given there has been no commensurate revision of the perceived risk of renal injury, conservatism bias is maintained. Both the incidence of AKI and frequency of contrast used in the critically ill remain high [19], but there is a higher probability that renal injury is due to other etiologies (e.g., sepsis, medications, and volume overload) [18]. Tests should be performed with a clear analysis of the risks and benefits. Modern contrast is no longer hyperosmolar; the risk for AKI is likely low. Therefore, the better question is whether the addition of contrast will help change management. It is time for a revision of beliefs concerning contrast as a nephrotoxic agent. 

Volume tolerance: automation bias (algorithms and automation trump clinical judgment)

Assessment of volume status is a crucial part of the evaluation of a critically ill patient. Both volume depletion and volume overload can lead to complications, but most algorithms (whether they are mandated or internal) favor volume administration [18]. Patients presenting to the emergency department or ICU suspected of sepsis will receive 30 ccs/kg of fluid, sometimes without deliberation of the patient’s tolerance to fluids. We fall prey to the automation bias. Most patients will tolerate the sepsis bolus, but as intensivists with the ability to use ultrasound, we can identify the patients that will be harmed by large fluid bolus. Volume tolerance can be summarized in one question: “Will I cause harm if I administer fluids to my patient?” Patients with pulmonary edema, right ventricular dysfunction, or IAH would be volume intolerant as fluids would likely exacerbate the clinical scenario. Ultrasound is a quick, inexpensive method to assess for volume tolerance, and should be incorporated into any decision to give fluids in the critically ill. Protocols have an essential role in the ICU, but vigilance needs to be maintained to identify potential patient harm by automation and algorithms. 

## Conclusions

The ICU is an extraordinarily complex environment which can be cognitively taxing for intensivists. When difficult decision making is frequent and sustained, intensivists are predisposed to cognitive biases which can lead to poor outcomes for patients. Addressing the problem of cognitive biases in ICU requires intellectual humility to recognize the fallibility of the human mind, paving the way for metacognition. We propose the use of metacognition in the ICU for a higher vantage point to view our errors in logic, which can only benefit our patients.
